# Plasma Hemopexin ameliorates murine spinal cord injury by switching microglia from the M1 state to the M2 state

**DOI:** 10.1038/s41419-017-0236-8

**Published:** 2018-02-07

**Authors:** Dunxin Han, Zhongwang Yu, Weili Liu, Dou Yin, Yingyan Pu, Jifeng Feng, Yimin Yuan, Aijun Huang, Li Cao, Cheng He

**Affiliations:** 1 Institute of Neuroscience and Key Laboratory of Molecular Neurobiology of Ministry of Education, Second Military Medical University, 200433 Shanghai, China; 2 Department of Spine Surgery, 107th Hospital of People’s Liberation Army, 264002 Yantai, China; 3 Institute of Physical Science and Information Technology, Anhui University, 230601 Hefei, China

## Abstract

Spinal cord injury (SCI) is a devastating type of central nervous system (CNS) trauma with limited therapeutic treatments. The polarization of microglia into the M1 or M2 state has been documented to play important roles in the pathogenesis of SCI, although the complete repertoire of underlying factors has not been identified. Interestingly, the time point at which hematomyelia (intramedullary spinal cord hemorrhage) is alleviated coincides with a decrease in the number of M2 microglia. Here the function of Hemopexin (Hpx), a hematogenous glycoprotein, was examined in the crush model of SCI. Hpx levels were elevated at the lesion site during hematomyelia and were synchronously correlated with the level of the M2 marker Arginase-1 (Arg-1). Ablation of Hpx in vivo affected the polarization state of lipopolysaccharide (LPS)-stimulated microglia, as mirrored by a lower percentage of M2 microglia and a higher percentage of M1 microglia in the lesion site, which delayed the recovery and exacerbated the behavioral dysfunction after SCI. However, Hpx induced a rapid switch from the M1 to M2 phenotype in LPS-stimulated primary cultured microglia in a heme scavenging-independent manner. The supernant of Hpx-treated microglia ameliorated neuronal degeneration, alleviated demyelination, and promoted oligodendrocyte precursor cell (OPC) maturation. This modulatory effect of Hpx on microglia polarization was at least partially mediated by the LRP-1 receptor. Based on these results, Hpx is considered a novel modulator of the polarization of microglia during the pathogenesis of SCI and may play a crucial role in the recovery from SCI.

## Introduction

Central nervous system (CNS) trauma, particularly SCI, is a major global challenge, with high mortality and disability rates. During SCI, the initial insult triggers a complex local inflammatory response, thus causing a secondary attack that results in a self-destroying cascade with more severe demyelination and neurodegeneration. Since the initial loss of neurons caused by primary injury is inevitable, most therapeutic strategies for CNS injury are based on reducing the deleterious effects of the secondary injury.

Microglia, which have long been considered one of the earliest and important participants in neuroinflammation in the CNS^1,2^, elicit detrimental or beneficial effects on remyelination and neural regeneration^3–5^. These divergent effects might be attributed to distinct microglial subsets and polarization states present in a dynamic equilibrium, such as the pro-inflammatory (M1) or anti-inflammatory (M2) states^6,7^. Microglia polarization is induced by different factors and plays distinct roles during the pathogenesis of SCI. For example, inducible nitric oxide (iNOS) induces M1 polarization of macrophages in the rat model of SCI^8^. Tumor necrosis factor (TNF) and iron induce the macrophage M1 phenotype in the injured spinal cord^9^. RGMa (repulsive guidance molecule a) is a potent inhibitor of axon regeneration by promoting M1 polarization^10^. Moreover, as shown in the study by Bartus K et al., chondroitin sulfate proteoglycan administration inhibits repair by promoting M1 polarization^11^. However, although more factors are required for M1 polarization, few factors have been identified to induce M2 polarization after SCI.

The time periods in which the polarized microglia are detected in the CNS after primary SCI differ. Although the M1 microglia response is rapidly induced and sustained following injury, the M2 cells are transiently increased within a 1 week post-lesion period and progressively decrease thereafter^12^. However, the identity of the intrinsic factors that steer the transient M2 polarization after SCI remains unclear. Although the question of whether the transient M2 polarization is a self-limited reaction that is beneficial for regeneration or just a recovery failure remains to be settled, microglia polarization appears to depend on signals in the lesion microenvironment. Vascular lesions and bleeding are the earliest environmental changes after injury, and abnormal increases in vascular permeability result in further production of protein-rich exudates, leading to local edema. The integrity of the blood–brain barrier starts to be repaired and edema begins to be resolved after 7 days^13^. Interestingly, the numbers of M2-polarized microglia begin to decrease at the same time point^12,14^. Therefore, we hypothesized that some hematogenous factors may exert essential roles in regulating the M2 polarization of microglia after SCI.

Hemopexin (Hpx), an acute-phase plasma glycoprotein with an extremely high binding affinity for heme, is responsible for reducing heme toxicity by transporting free heme to intracellular compartments, preventing it from generating free radical reactions^15^. In adults, serum Hpx concentrations range from 0.40 to 1.50 g/L^16^. Hpx is mainly synthesized by hepatic cells and is also expressed in all regions of the CNS^17^. In addition, Hpx levels are also remarkably increased under several pathological conditions, such as peripheral neuron degeneration, cerebral ischemia, and SCI^18,19^. Despite this new knowledge, the function of Hpx and the underlying mechanism remain to be fully defined.

In the present study, we examined whether Hpx participates in the polarization of microglia after SCI. Hpx was required to switch microglia polarization from the lipopolysaccharide (LPS)-induced M1 phenotype to the M2 phenotype, and ablation of Hpx in vivo inhibited the M2 polarization of microglia, aggravated the pathology of trauma, and prohibited recovery from SCI.

## Results

### Hpx expression in the spinal cord is dynamically regulated following crush injury

**Fig. 1:**
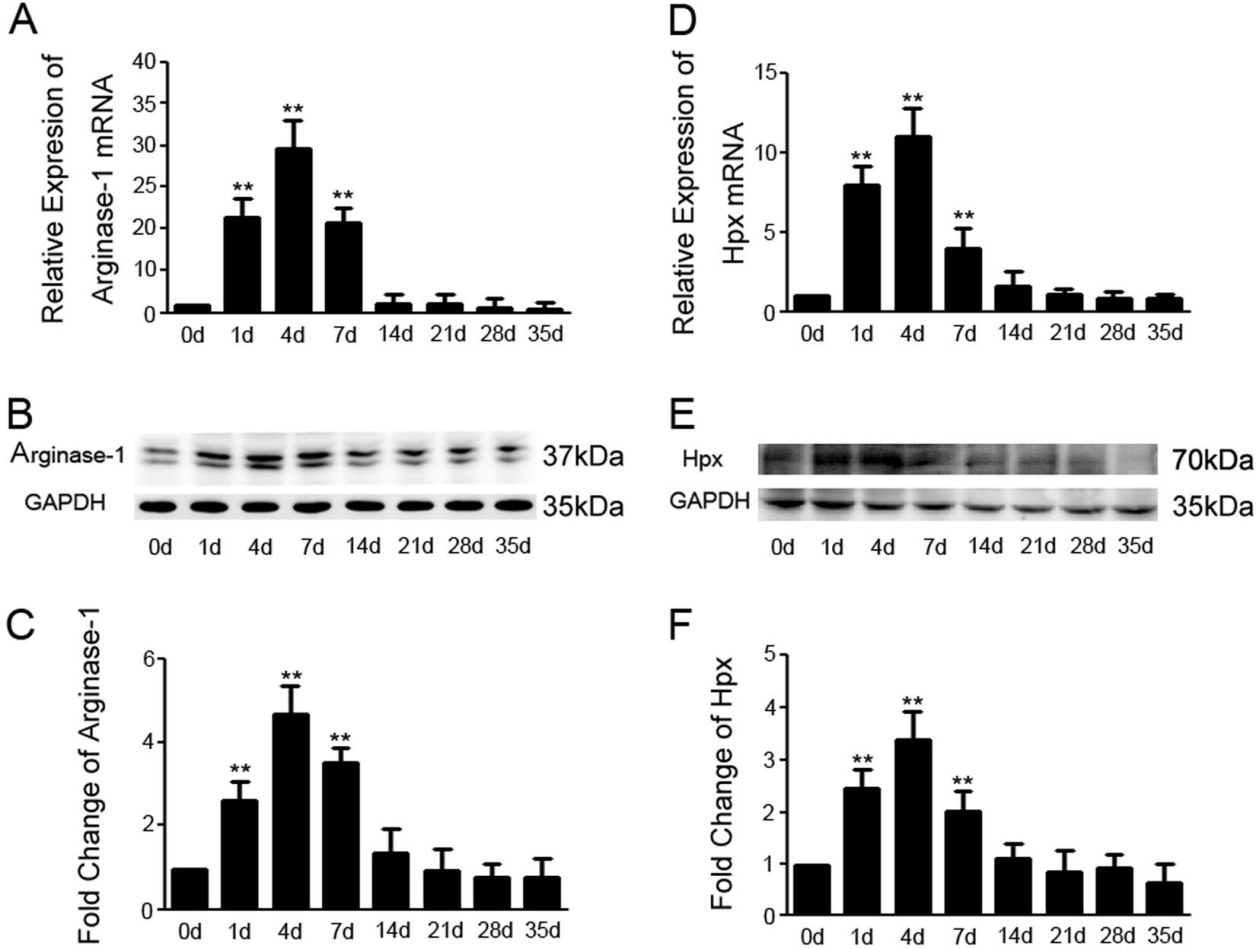
Hemopexin (Hpx) and Arginase-1 (Arg-1) share similar expression patterns in the mouse spinal cord after SCI. **a**, **d** Relative expression of the Arg-1 and Hpx mRNAs in the mouse spinal cord at the indicated time points post-lesion. **b**,** c**,** e**, **f** Expression of the Hpx and Arg-1 proteins in the mouse spinal cord post-lesion. *p < 0.05, **p < 0.01 compared with the control mice. *N* = 24 mice per group, with three mice at each time point. AU arbitrary units. The data are presented as the means ± SEM of three independent experiments

We first examined the expression of Hpx and Arg-1, a typical M2 marker, in the lesioned site of the spinal cord by quantitative PCR (qPCR) and immunoblotting at 1, 4, 7, 14, 21, 28, and 35 days post lesion (dpl). Two-millimeter sections of the spinal cord adjacent to the injury epicenter were collected to examine the expression of the Arg-1 and Hpx mRNAs. Compared with the sham group (0 dpl), the levels of both the Hpx and Arg-1 mRNAs were simultaneously increased at 1 week post-lesion (1, 4, and 7 dpl) and reduced to levels comparable to the control at 2 weeks post-lesion (14, 21, 28, and 35 dpl) (Fig. 1a, d). An immunoblot analysis on the protein levels also illustrated that the levels of both the Hpx and Arg-1 proteins were increased early after SCI and simultaneously reduced 2 weeks post-lesion (Fig. 1b, c, e, f). Thus, consistent with the expression pattern of the M2 marker Arg-1, Hpx expression was dramatically increased during the early stage of SCI.

### Microglia in Hpx^−/−^ mice prominently display an M1-dominant phenotype following crush injury

**Fig. 2:**
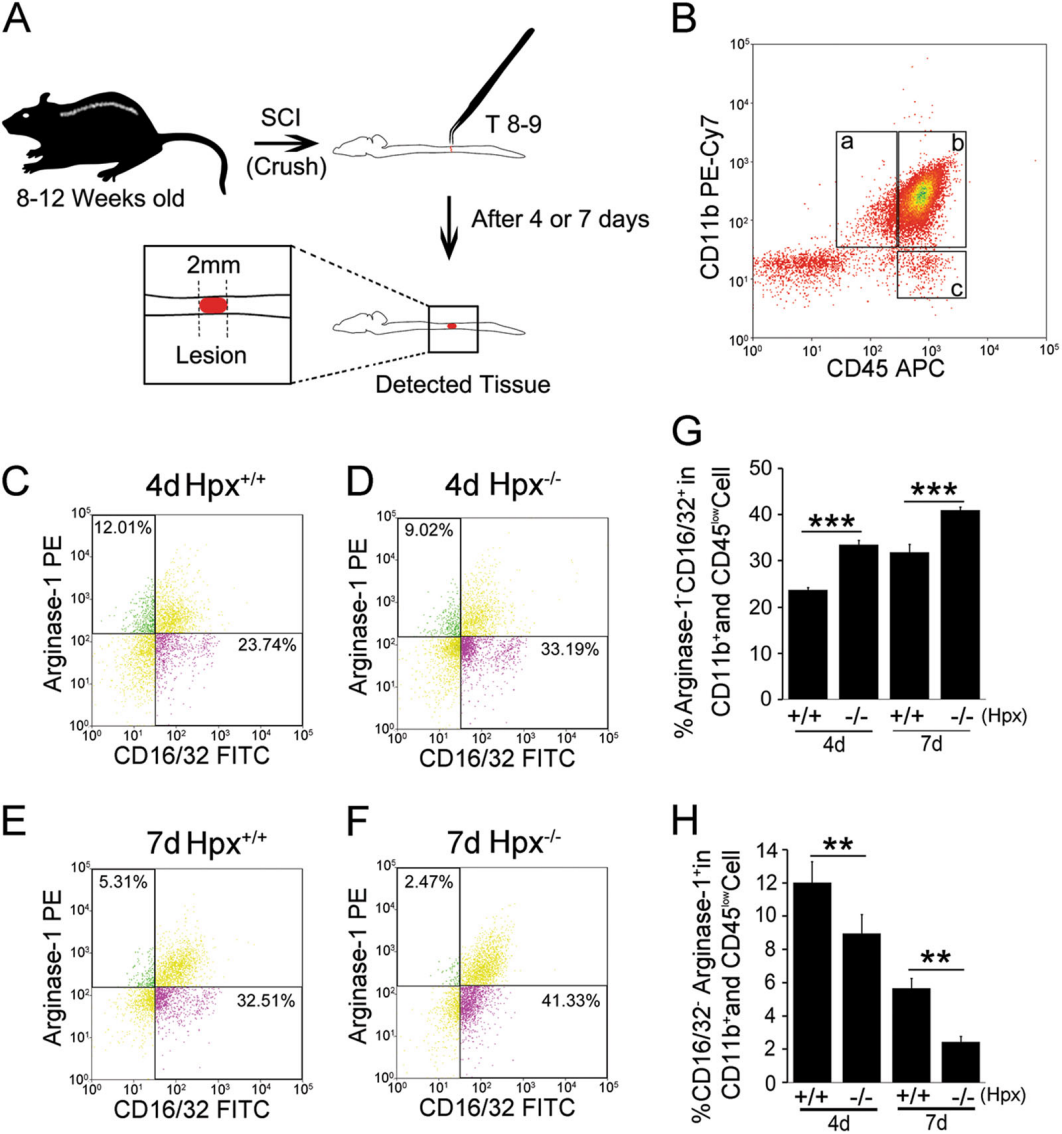
The percentages of M1 and M2 polarized microglia after spinal cord injury. **a** Schematic diagram of the SCI model. The crushed SCI was induced at T8–T9 of the spinal cord in adult mice (8–12 weeks). The indicated tissue was then detected on 4 or 7 dpl. **b**–**f** Representative micrographs showing the flow cytometric analysis of microglia isolated from the spinal cords of Hemopexin knockout mice (Hpx^−/−^) and control mice (Hpx^+/+^) on 4 or 7 dpl. **b** Flow cytometric analysis of microglia isolated from the mouse spinal cord. Mononuclear cells were isolated, stained for CD11b and CD45, CD16/32, and Arg-1, and then analyzed using FACS. CD11b^+^CD45^low^ resting microglia (a), CD11b^+^CD45^hi^ activated microglia and peripheral macrophages (b), and CD11b^+^CD45^hi^ lymphocytes (c) are shown. **g**, **h** Percentage of M1 (CD16/32^+^Arg-1^−^CD11b^+^CD45^low^) and M2 (CD16/32^−^Arg-1^+^CD11b^+^CD45^low^) polarized microglia from the spinal cord of Hpx^−/−^ and Hpx^+/+^ mice on day 4 or 7 post spinal cord crush lesion. Hpx^−/−^ and Hpx^+/+^ mice were subjected to a mid-thoracic (T8–T9) laminectomy or SCI. The data are presented as the means ± SEM of three independent experiments, *n* = 5 per group at each time point. **p < 0.01, ***p < 0.005 compared with the indicated control

We then assessed the polarization of microglia using a fluorescence-activated cell sorting (FACS) analysis. The potency of polarized CD45^low^/CD11b^+^ microglia (Fig. 2a, b) enriched from spinal cord was analyzed 4 or 7 days post crush injury. Studies of microglia in the spinal cord of Hpx^−/−^ mice with polarization state-specific markers revealed a shift from an M2- to an M1-dominant phenotype following the initiation of crush injury. On 4 dpl, substantially more CD45^low^/CD11b^+^ microglia from Hpx^−/−^ mice expressed the M1 marker CD16/32 than microglia from Hpx^+/+^ mice (Fig. 2c, d, g), whereas relatively fewer CD45^low^/CD11b^+^ microglia from Hpx^−/−^ mice expressed the M2 marker Arg-1 than microglia from Hpx^+/+^ mice (Fig. 2c, d, h). Accordingly, on 7 dpl, still more CD45^low^/CD11b^+^ microglia from Hpx^−/−^ mice expressed the M1 marker CD16/32 than microglia from the Hpx^+/+^ mice (Fig. 2e, f, g), whereas even fewer CD45^low^/CD11b^+^ microglia expressed the M2 marker Arg-1 than the Hpx^+/+^ mice (Fig. 2e, f, h). In summary, microglia in the Hpx^−/−^ mice prominently displayed an M1-dominant phenotype following crush injury.

**Fig. 3:**
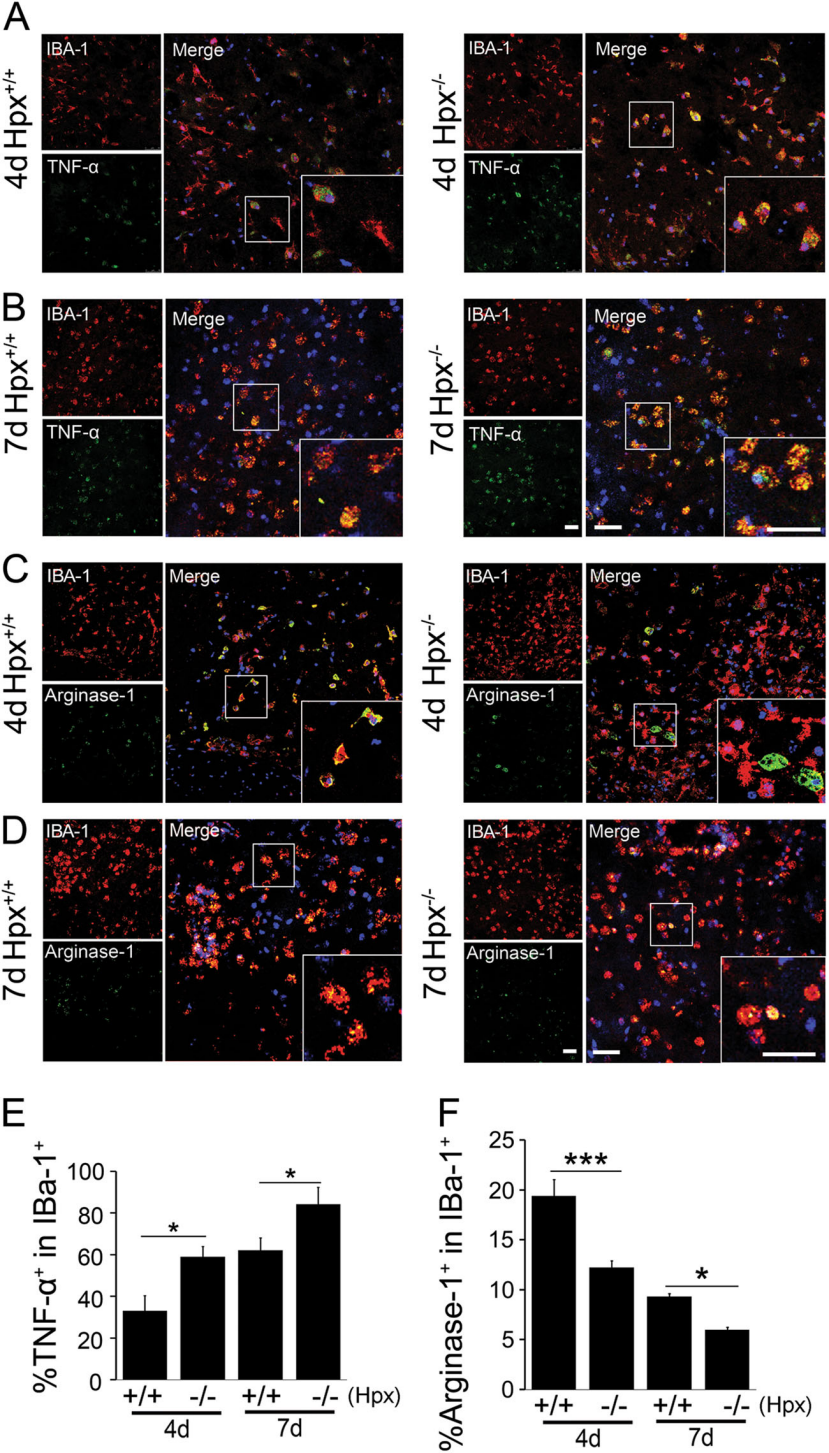
Numbers of TNF-α^+^/IBA-1^+^ microglia are increased, while numbers of Arg-1^+^/IBA-1^+^ microglia are decreased, within the lesion sites of spinal cords of Hpx^−/−^ mice after spinal cord injury. **a**–**d** Representative images of TNF-α^+^/IBA-1^+^ (**a**, **b**) or Arg-1_+_/IBA-1^+^ (**c**, **d**) microglia in the spinal cord after crush injury on 4 (**a**, **c**) and 7 (**b**, **d**) dpl. **e**, **f** Quantification of the number of TNF-α^+^/ IBA-1^+^ (**e**) or Arg-1^+^/IBA-1^+^ (**f**) microglia in spinal cords from Hpx^−/−^ and Hpx^+/+^ mice after crush injury. *p < 0.05, ***p < 0.005 compared with the indicated control. Scale bar = 30 µm. *N* = 6 mice per group at each time point

We also quantified the numbers of M1 and M2 microglia within the lesion sites of spinal cords after SCI by immunofluorescence staining with TNF-α (M1) and Arg-1 (M2) antibodies, respectively. On both 4 and 7 dpl, the percentages of TNF-α^+^/IBA-1^+^ microglia were robustly increased in Hpx^−/−^ mice compared with Hpx^+/+^ mice (Fig. 3). In contrast, the percentages of Arg-1^+^/IBA-1^+^ microglia were significantly decreased in Hpx^−/−^ mice compared with Hpx^+/+^ mice on both 4 and 7 dpl (Fig. 3). These results consistently indicate a bias toward the M1-polarized microglia in Hpx^−/−^ mice following SCI.

Generally, microglia polarization can switch between the M1 and M2 phenotype, depending on the microenvironment. LPS-pretreated microglia, which were confirmed to have the M1 phenotype in vitro, were transplanted into the T9 segment spinal cord of Hpx^−/−^ or Hpx^+/+^ mice and the Arg-1^+^/GFP^+^ microglia were examined in situ 3 dpl using immunofluorescence staining to examine the effect of Hpx on microglia polarization in vivo. The quantification the percentage of Arg-1^+^/GFP^+^ microglia illustrated that fewer M1 microglia switched to the M2 phenotype in Hpx^−/−^ mice on 3 dpl (Supplementary Figure 1). Based on this result, Hpx played an essential role in promoting the switch from the M1 phenotype to the M2 phenotype.

### Hpx^−/−^ mice show impaired functional recovery and aggravated lesion after SCI

**Fig. 4:**
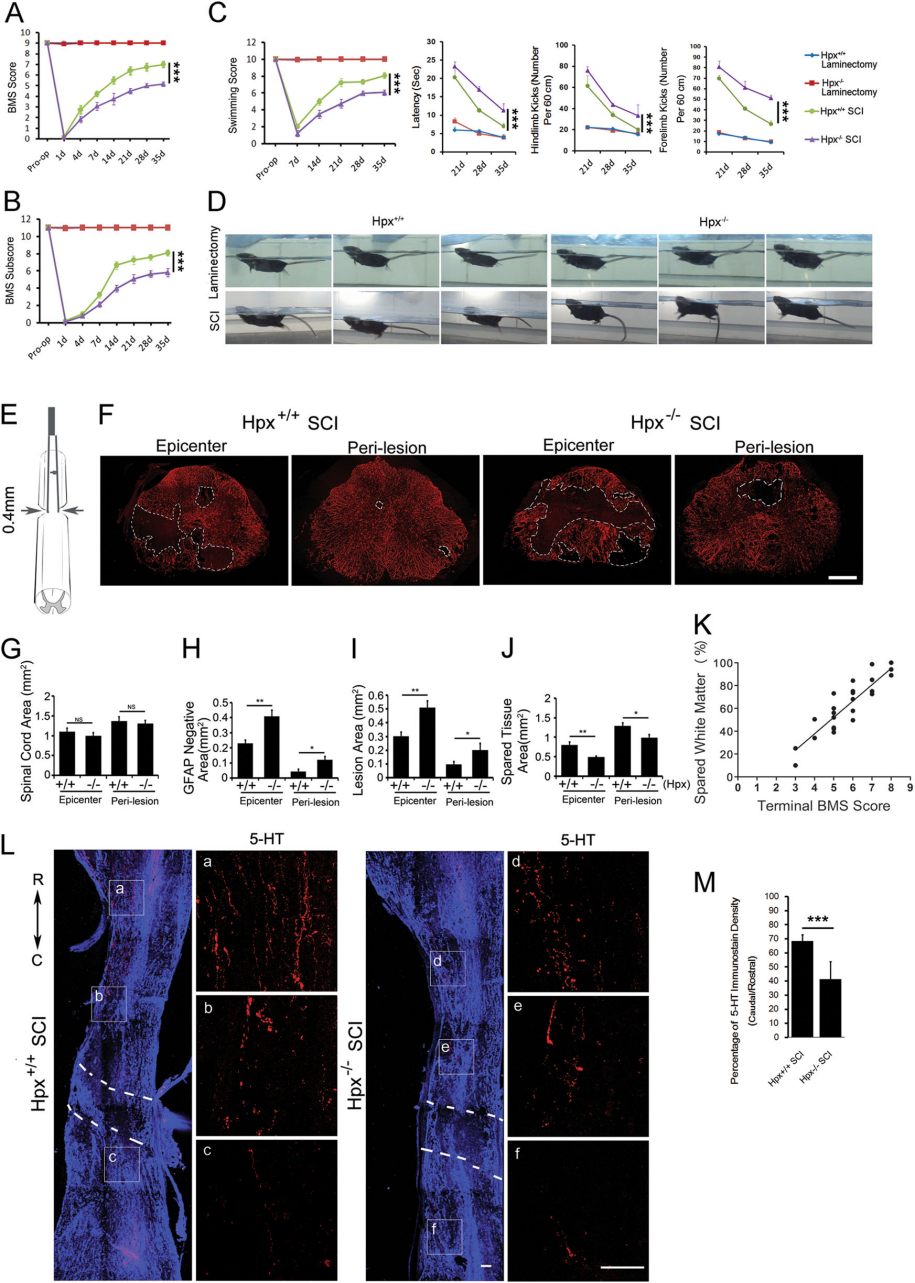
Hemopexin knockout mice showed reduced functional recovery of hindlimb after SCI. Functional recovery was assessed by determining the Basso Mouse Scale (BMS) scores (**a**), Basso Mouse Scale subscores (**b**) and swimming tests (**c**, **d**) over a 35-day period (*n* = 15 mice per group). **e** Schematic graph of the thoracic spinal cord (T8–T9) crush model used. **f** Representative images of GFAP immunostaining in spinal cord sections at the epicenter and peri-lesion sites from Hpx^−/−^ and Hpx^+/+^ mice on 35 dpl after SCI. The white line denotes the size of the lesion. Quantification of GFAP immunostaining in the total tissue area (**g**), GFAP-negative area (**h**), lesion area (**i**), and spared tissue (**j**). The relationship between spared white matter and final BMS score of each mouse was assessed with Pearson’s regression analysis (**k**). **l** Representative images of 5-HT^+^ fibers (red) co-stained with GFAP (blue) in sagittal sections. **m** Quantification reveals a significant decrease in 5-HT^+^ fiber sprouting caudal to the injury in Hpx^−/−^ SCI group vs. Hpx^+/+^ SCI group mice on 35 dpl. **a**–**f** 5-HT^+^ fibers in the boxed areas are enlarged in the right panels in two groups. Dashed lines indicate lesion margins. (*n* = 5 mice per group). *p < 0.05, **p < 0.01, ***p < 0.005, compared with the indicated control. Scale bar = 100 µm

We assessed and compared the functional recovery between Hpx^−/−^ and Hpx^+/+^ mice to further determine the function of Hpx depletion during SCI. Mice were assessed using the Basso Mouse Scale (BMS) scores, BMS subscores, and swimming scores (Fig. 4). The Hpx^−/−^ SCI group showed lower BMS scores, BMS subscores, and swimming scores than the Hpx^+/+^ SCI group beginning on 7 dpl (Fig. 4a–c), which was mirrored by tail-down body angle and variable rotation along the long axis of the body (Fig. 4d)^20^. Accordingly, longer time to reach the platform, more strokes of both forelimb and hindlimb were also observed in mice of the Hpx^−/−^ SCI group (Fig. 4c).

Stereological quantification of serial spinal cord sections by glial fibrillary acidic protein (GFAP) immunostaining (Fig. 4e, f) revealed that the lesion area (Fig. 4i), including the GFAP^−^ area (Fig. 4h) and cavities^21^, was larger in the peri-lesion and epicenter regions of the Hpx^−/−^ SCI group than in the Hpx^+/+^ SCI group, whereas the spared tissue area was smaller in the Hpx^−/−^ SCI group than in the Hpx^+/+^ SCI group (Fig. 4j). No significant difference in the total area was observed between these groups (Fig. 4g).

According to Luxol fast blue (LFB) staining, myelinated areas in the spinal cords of Hpx^−/−^ mice were significantly smaller than the myelinated areas in Hpx^+/+^ mice on 7 dpl. Quantification of the dorsal spinal cord in Hpx^−/−^ and Hpx^+/+^ mice revealed that a lower percentage of spared areas in the total white matter was observed in both the epicenter and peri-lesion sites of Hpx^−/−^ mice than in Hpx^+/+^ mice (Supplementary Figure 2).

To detect whether the rate and extent of recovery correlated with the area of spared tissue, the relationship between spared white matter and final BMS score of each mouse was assessed with Pearson’s regression analysis. The percentage of spared white matter significantly correlated with BMS scores (Fig. 4k).

Furthermore, quantification of 5-HT^+^ fibers indicated that spared serotonergic fibers were rarely detected at caudal regions in Hpx^−/−^ SCI group (Fig. 4l). By contrast, more serotonergic fibers were detected at caudal regions in the Hpx^+/+^ SCI group (Fig. 4l, m).

Moreover, based on the quantification of NeuN^+^ neurons, significantly fewer neurons survived in the epicenter and peri-lesion sites of the spinal cord of Hpx^−/−^ mice after SCI than in Hpx^+/+^ mice, particularly in the ventral horn (Supplementary Figure 3).

### Hpx switches M1 microglia to the M2 polarization state in vitro

**Fig. 5:**
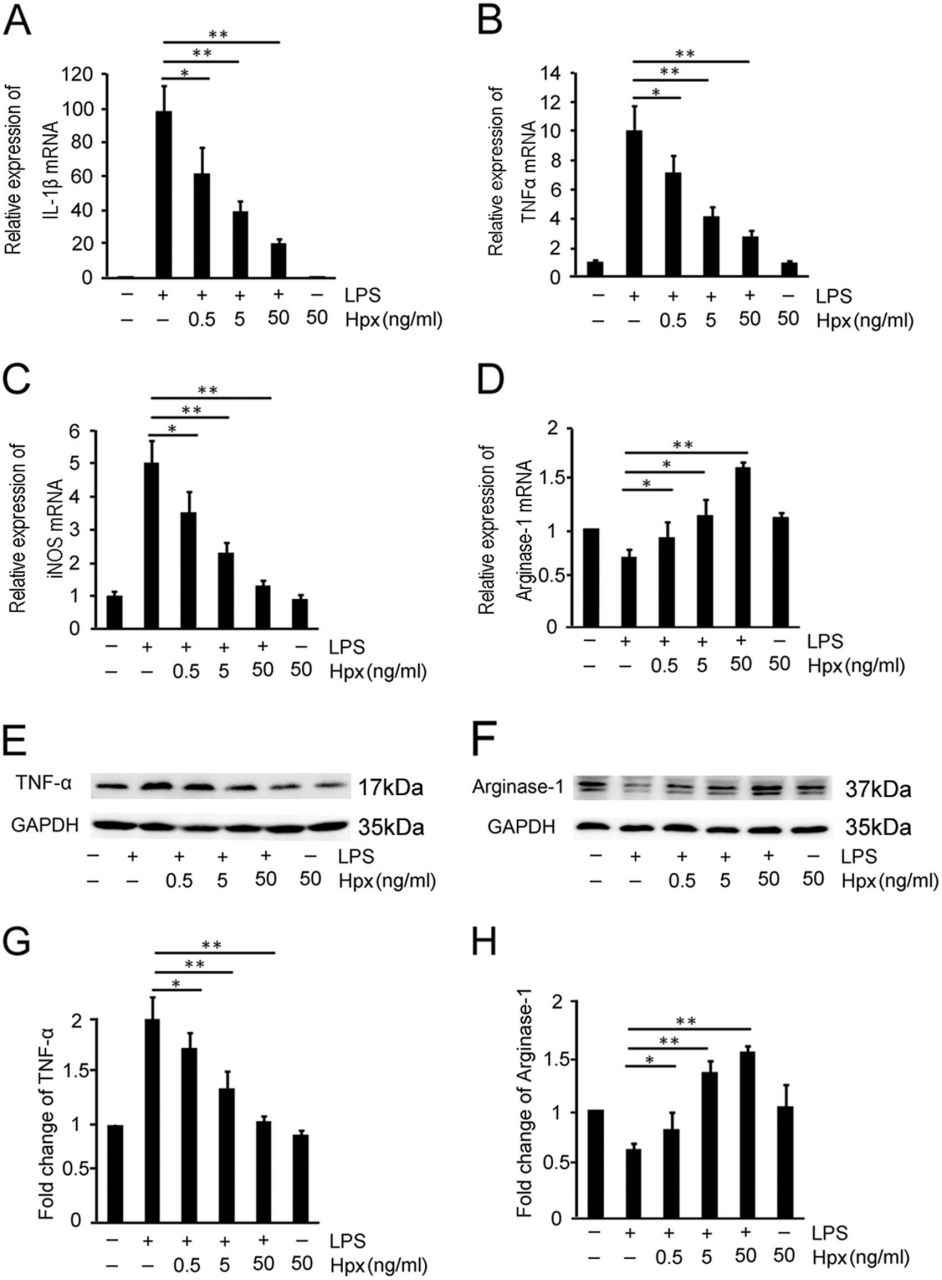
Hpx switches M1 microglia to the M2 polarization state in vitro. **a**–**d** Effects of Hpx on the polarization of LPS-stimulated microglia (MG^LPS^) were determined using quantitative RT-PCR. IL-1β, iNOS, and TNF-α were used as M1 markers (**a**–**c**) and Arg-1 was used as an M2 marker (**d**). **e**–**h** Western blot analysis of TNF-α (**e**) and Arg-1 (**f**) expression in LPS-stimulated microglia. **g**,** h** Scanning densitometry of TNF–α (**g**) and Arg-1 (**h**) levels, which were quantified and normalized to GAPDH. *p < 0.05, **p < 0.01 compared with the indicated control. Data are presented as the means ± SEM of at least three independent experiments

The above observations prompted us to explore whether Hpx directly induced an M1/M2 phenotype switch in microglia. Arg-1 was used as a marker of M2 polarization, whereas interleukin (IL)-1β, iNOS, and TNF-α were used to define M1 polarization. Hpx counteracted the LPS-induced downregulation of the Arg-1 mRNA and upregulation of IL-1β, iNOS, and TNF-α mRNAs in microglia in a dose-dependent manner (Fig. 5a–d). According to the results of the western blot analysis, Arg-1 levels were increased in Hpx-treated microglia, whereas TNF-α levels were decreased in response to the Hpx treatment (Fig. 5e–h). Notably, Hpx did not affect the expression of Arg-1, IL-1β, iNOS, or TNF-α in microglia that had not been pretreated with LPS (Fig. 5).

**Fig. 6:**
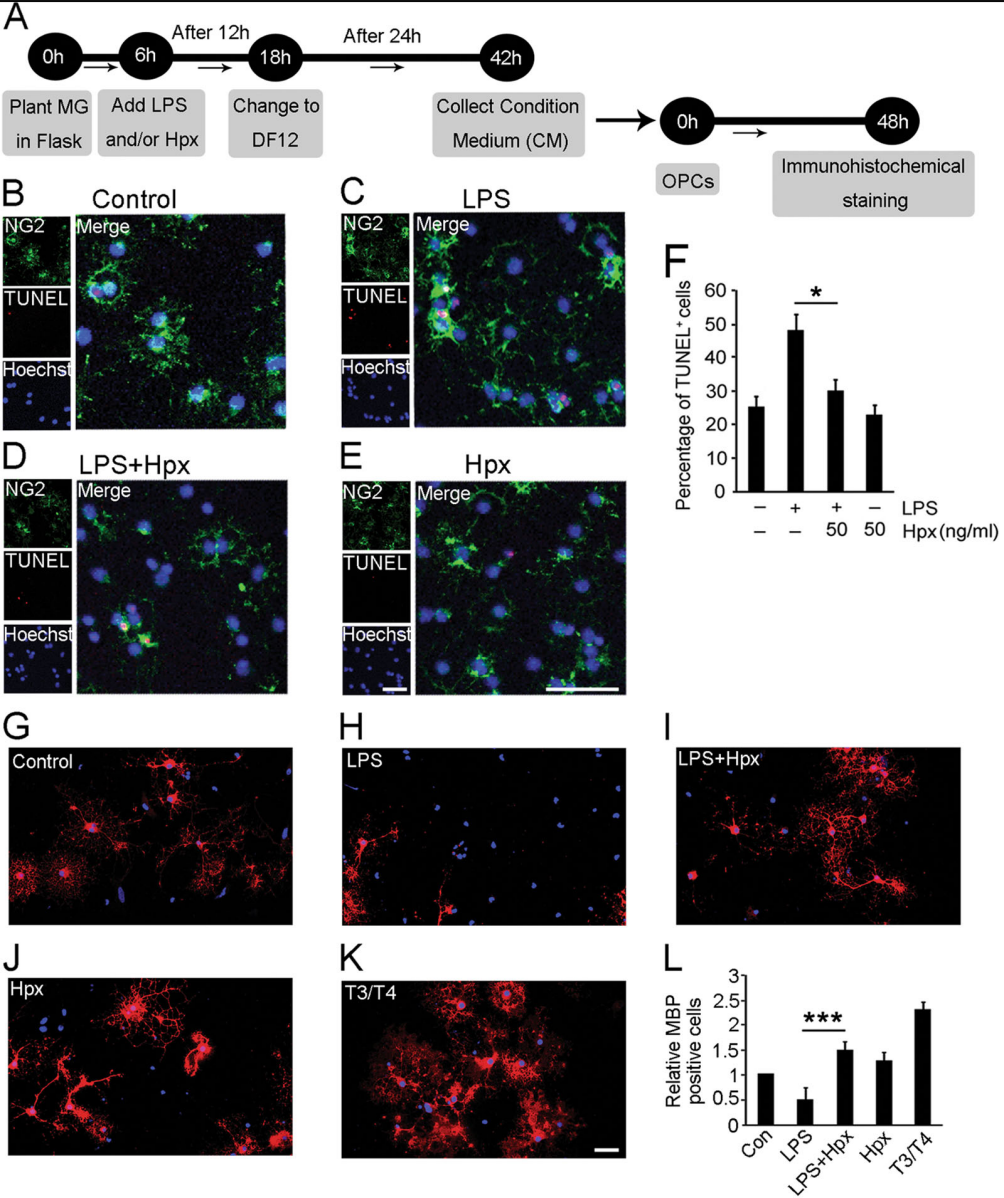
Hpx-induced microglia promoted the viability and the maturation of cultured OPCs. **a**–**e** OPCs were incubated with conditioned medium (CM) from MG^LPS^, MG^LPS+HPX^, MG^Hpx^, or unstimulated microglia (control) for 48 h and then analyzed using the TUNEL assay. **f** Quantification of the percentage of apoptotic cultured OPCs using the TUNEL assay. Scale bar = 40 µm. **g**–**k** Effects of the CM from microglia pretreated with the indicated compounds. Effects of Hpx-induced microglia pretreated with or without LPS on OPC differentiation were determined using an anti-MBP antibody (red). Control, not stimulated; triiodothyronine/thyroxine (T3/T4), positive control. **l** Histogram showing the percentage of MBP+ OPCs. Data are presented as the means ± SEM of three independent experiments. *p < 0.05, ***p < 0.005 compared with the indicated control. Scale bar = 25 µm

We further explored whether Hpx-treated microglia affected the viability of cultured neurons and oligodendrocyte precursor cells (OPCs) in vitro. The conditioned medium (CM) from the MG^LPS^ group dramatically increased the apoptosis of cultured neurons. In contrast, compared with the MG^LPS^ group, the percentages of apoptotic neurons decreased significantly in the MG^LPS+Hpx^ group (Supplementary Figure 4). Similarly, the percentages of apoptotic OPCs also decreased in the MG^LPS+Hpx^ group (Fig. 6). No significant apoptosis of cultured neurons or OPCs was observed in the MG^Hpx^ group.

We also investigated whether the Hpx treatment of microglia affected the maturation of OPCs. As shown in Fig. 6, the CM from MG^LPS+HPX^ promoted OPC maturation by increasing the number of MBP^+^ cells, whereas the CM from MG^LPS^ prohibited OPC maturation (Fig. 6). We did not observe a remarkable promotion of OPC maturation in the MG^Hpx^ group. Based on our results, Hpx-induced M2 polarization of microglia preserved the viability of neurons and OPCs and promoted the maturation of OPCs.

### Hpx regulates microglia polarization through low-density lipoprotein receptor-related protein (LRP)-1 receptor

**Fig. 7:**
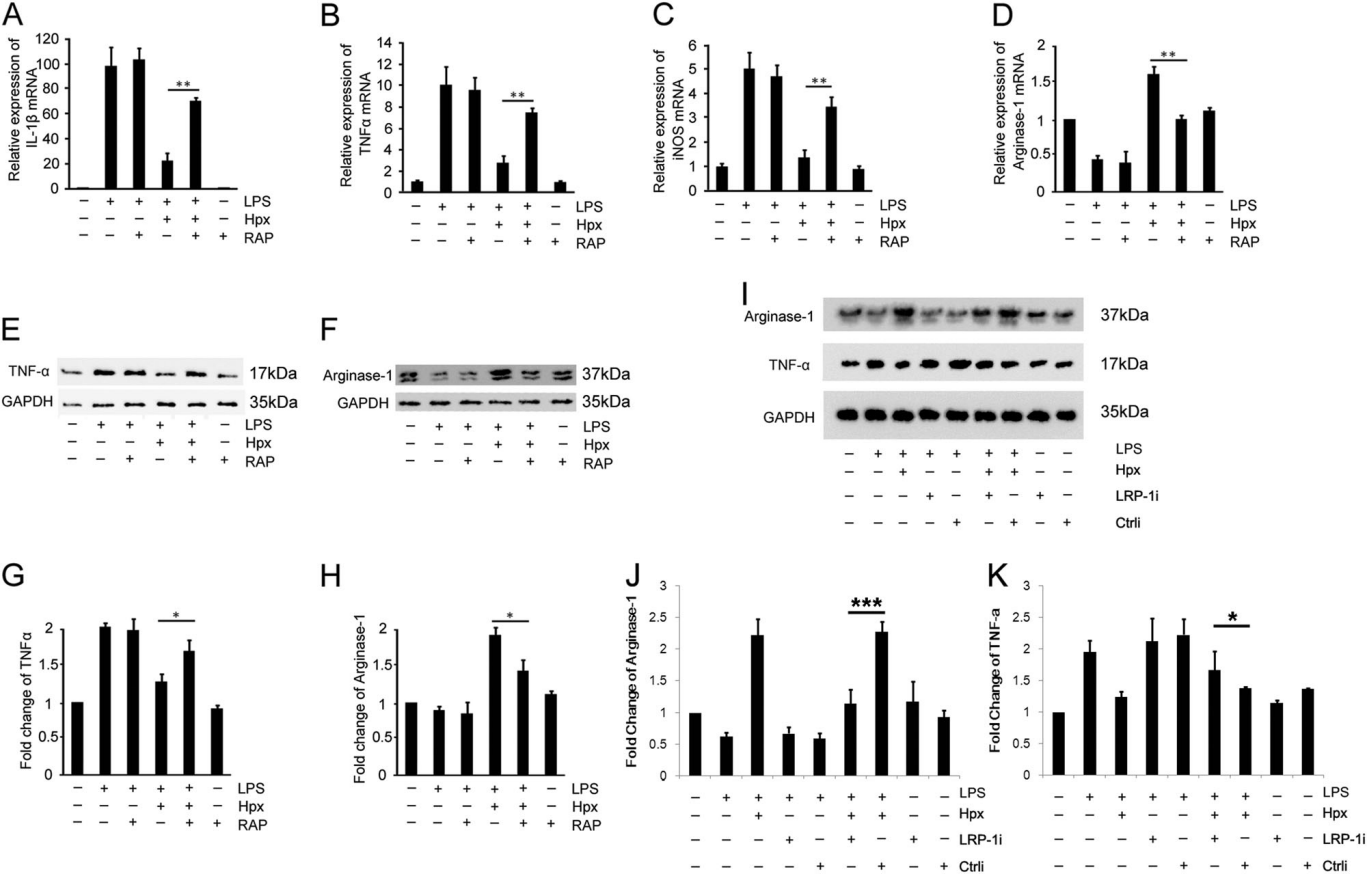
Hpx regulated microglia polarization via an LRP-1-dependent pathway. **a**–**d** Effects of Hpx on the polarization of LPS-stimulated microglia were determined using quantitative RT-PCR. IL-1β, iNOS, and TNF-α were used as M1 markers (**a**–**c**) and Arg-1 was used as an M2 marker (**d**). **e**–**k** Scanning densitometry of TNF-α (**e**,** g**,** i**, **k**) and Arg-1 (**f**,** h**,** i**, **j**) levels, which were quantified and normalized to GAPDH. Data are presented as the means ± SEM of at least three independent experiments. *p < 0.05, **p < 0.01, ***p < 0.005

According to previous studies^22,23^, LRP-1 is an Hpx receptor and is antagonized by receptor-associated protein (RAP) in vitro. We then investigated whether Hpx induced the M1/M2 switch in microglia polarization through the LRP-1 receptor. As mentioned above, Arg-1 was used as a marker of M2 polarization, whereas IL-1β, iNOS, and TNF-α were used to define M1 polarization. As shown in Fig. 7, the Hpx-mediated reversal of the LPS-induced downregulation of the M2 marker mRNA and upregulation of the M1 polarization mRNAs was abrogated by RAP or silencing LRP-1. Notably, RAP had no effect on the LPS-induced regulation of polarization marker expression in microglia without Hpx treatment. Thus Hpx switched M1 microglia to the M2 polarization state via an LRP-1-dependent pathway.

### Hpx promoted functional recovery and raphespinal sprouting in lesion spinal cord

**Fig. 8:**
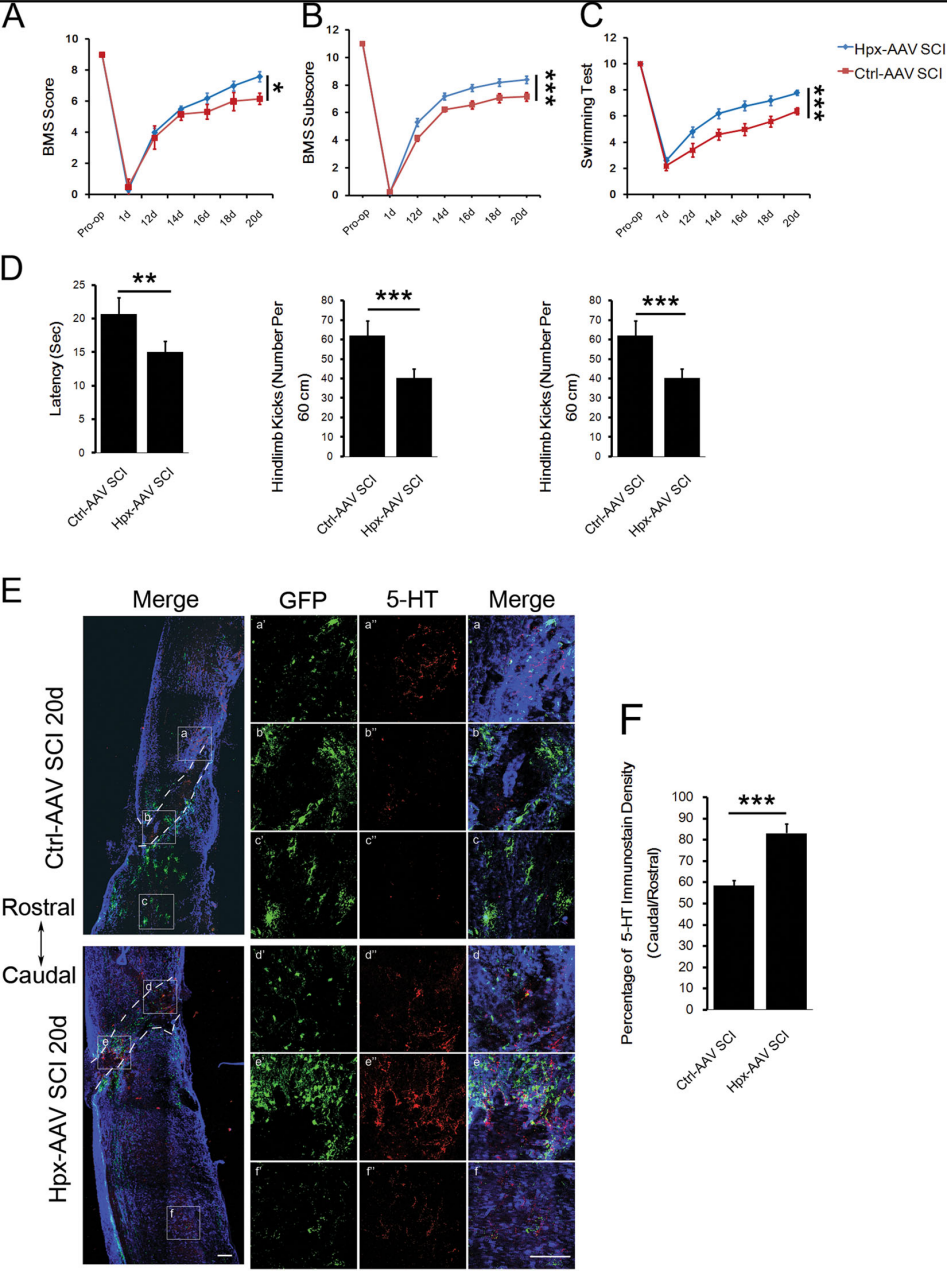
Hpx promoted functional recovery and raphespinal sprouting in lesion spinal cord of the Hpx-AAV SCI group. Functional recovery was assessed by determining BMS scores (**a**), BMS subscores (**b**), and swimming tests (**c**,** d**) between 2 and 3 weeks postsugery (*N* = 10 mice per group). **e** Representative images of 5-HT^+^ serotonergic fibers (red) in the middle panels, costained with GFAP (blue) and GFP (green) in sagittal sections. Green indicates expression of GFP fused with or without Hpx expressed by AAV vectors injected into the spinal cord at the lesion. a–f Boxed areas are enlarged in the right panels (a’–f’, a”–f”). **f** Quantification reveals a significant increase in 5-HT^+^ fiber sprouting rostral to the injury in the Hpx-AAV SCI group vs. Ctrl-AAV SCI group of mice on 20 dpl. Dashed lines indicate lesion margins. Scale bar = 100 μm; *p < 0.05, **p < 0.01, ***p < 0.005

To further determine whether Hpx could promote recovery or repair after SCI, mice of the Hpx-AAV SCI group or control group (Ctrl-AAV SCI) were assessed using the BMS scores, BMS subscores, and swimming scores, and 5-hydroxytryptamine (5-HT) immunostainings were performed to show whether serotoninergic raphespinal sprouting was affected (Fig. 8). The Hpx-AAV SCI group showed higher BMS scores, BMS subscores, and swimming scores than the Ctrl-AAV SCI group beginning on 14 dpl (Fig. 8a–c). Less time to reach the platform and fewer strokes of both forelimb and hindlimb were also observed in mice of the Hpx-AAV SCI group (Fig. 8d). Though the 5-HT^+^ axons from the mouse of Ctrl-AAV SCI group were rarely detected to cross the border of the glial scar on 20 dpl, a number of 5-HT^+^ axons grew into the GFAP^−^ lesion area in the mice of the Hpx-AAV SCI group (Fig. 8e). Increase in serotonergic axon density was also observed in the caudal area from epicenter, surrounded by GFP^+^ Hpx overexpressed cells on 20 dpl (Fig. 8f), exhibiting an irregular growth trajectory, which seems regenerated fibers^24^. Thus delivery of Hpx could promote function recovery and axon repair after SCI.

## Discussion

Microglia are the functionally flexible first responders to CNS injury^9^. Although microglia predominantly retain the M1 phenotype, a synchronous upregulation of M2 phenotype markers was observed within the first week after SCI^12^. However, few explanations for the heterogeneous phenotypes of microglia at the lesion site have been proposed. Here we performed a study to examine the function of Hpx in the crush model of SCI and found that Hpx levels were elevated in the lesion site during hematomyelia. Moreover, Hpx switched M1 microglia to the M2 polarization state in vitro. Ablation of Hpx in vivo inhibited M2 microglia polarization, aggravated the pathology of trauma, and prohibited functional recovery, while high dose of Hpx promoted neuroprotection and function recovery after SCI.

Until recently, no hematogenous factors had been discerned to regulate the polarization of microglia. According to the study by Kroner et al, iron derived from senescent red blood cells induced a rapid switch from the M2 phenotype to the M1 phenotype following SCI^9^. Nevertheless, factors that induce the transient increase in the M2 polarization (decrease within 7 days) of microglia after SCI are still unknown. Phagocytosis of myelin and dying cells was reported to occur for at least 2 weeks after injury^9^, which promotes M2 polarization in vitro^25^. However, myelin debris at the lesion site was shown to switch macrophages in the spinal cord from the M2 phenotype to the M1 phenotype^26^. Thus the discrepancy in the effect of myelin debris on the polarization of microglia implies that other potential factors induce the transient increase of M2 polarization after lesion. The time course for the re-establishment of the blood–spinal cord barrier was reported to be no longer than 14 days after moderate injury^27^. The resolution of the initial hemorrhage occurred concomitantly with the decrease in the M2 phenotype of microglia over 7 days^27^. Therefore, Hpx, an acute-phase protein, may act as a hemorrhage-derived factor to transiently induce the M2 phenotype in microglia after the initial SCI until the recovery of integrity of the blood–spinal cord barrier.

Hpx, which has an extremely high binding affinity for heme, has been consistently shown to reduce heme toxicity by transporting free heme to intracellular compartments where it is catabolized by heme-oxygenase enzymes. The roles of Hpx in the regulation of inflammation are beginning to be appreciated. Tian L et al. identified Hpx as an anti-inflammatory factor that suppressed the synergistic actions of hemoglobin and high mobility group box 1 on the pro-inflammatory activation of macrophages^28^. Former studies reported that Hpx alleviates macrophage proinflammatory state in atherosclerosis and sickle cell disease^29,30^. Hpx was also shown to serve as a negative regulator of the Th17 response and alleviated the development of experimental autoimmune encephalomyelitis (EAE)^31^. Moreover, Hpx has been shown to downregulate LPS-induced TNF and IL-6 expression in macrophages during Gram-negative bacterial infections^32^. As shown in the recent study by Ma and colleagues, deletion of Hpx or heme oxygenase-2 aggravates brain injury after intracerebral hemorrhage^33^. Considering the time point at which hematomyelia is alleviated coincides with a decrease in the number of M2 microglia, we hypothesize that Hpx, a hematogenous glycoprotein and an anti-inflammatory factor in several disease, which was much higher in the blood than in the spinal cord, might affect microglia activation during hematomyelia. In the present study, we expanded the previous findings and showed that Hpx was sufficient to switch microglia polarization from the M1 phenotype to the M2 phenotype. Owing to the insufficient level of supporting microglia in the later stage of spinal cord insult and the lack of side effects of the Hpx treatment^34^, our results highlight the potential utility of Hpx or adoptive transfer of Hpx-induced microglia at the lesion site in a specific time frame to alleviate the secondary injury and improve functional repair after SCI.

Here we used a modified forceps crush model, which was first developed by Plemel et al. Relative low expensive equipments, simple surgeries fitting to mice, short time costs, relative consistent behavioral and pathologic outcome in mice, less disturbance to the fragile bony support as well as clinical relevance highlight the crush model as a useful tool on spinal cord research in mice^35^. Of course, the effect of Hpx on the recovery of SCI may also need to be determined carefully in other models, including the more clinically relevant contusive injury.

Heme has been shown to promote M1 polarization of microglia via Toll-like receptor 4, inducing activation of c-Jun N-terminal kinase and p38, increasing the levels of TNF, IL-6, and IL-1β, and consequently causing inflammatory injury^36^. Although Hpx directly promoted the switch from M1 to M2 polarization of microglia in vitro, Hpx may have also blocked the M1 phenotype of microglia in vivo through its heme-scavenging activity^30^. However, the heme-scavenging function of plasma Hpx is observed at very high protein concentrations (10–20 µM, ~ 1 g/L)^37^, whereas low levels of HPX (2.6 mg/L) are observed in the cerebrospinal fluid^19^. Actually, a range of 1.8–3.4 mg/L in the cerebrospinal fluid might be insufficient to cope with the quantities of heme released during ischemia and reperfusion^38^. In the present study, a lower level of Hpx (50 ng/ml) in the culture medium switched M1 microglia to the M2 phenotype in vitro. Based on the result from our transplantation experiment showing that M1 microglia were converted to the M2 phenotype in normal spinal cord where both Hpx and Heme are present at very low levels in situ, we plausibly inferred that Hpx blocked the M1 phenotype of microglia in vivo through a mechanism independent of its heme-scavenging activity.

Owing to the crucial role of Hpx in protecting myelin^39^, we should also consider the possibility that Hpx promotes the recovery of SCI by directly affecting the function of oligodendrocytes. Demyelination in Hpx^−/−^ mice with EAE was not only due to T lymphocyte infiltration but also to the impairment of oligodendrocytes^31^. In addition to the protective effects on heme-induced oxidative stress^17^, Hpx was reported to promote oligodendrocyte differentiation in the adult CNS after a demyelinating insult^39^. In the present study, the proportion of spared myelin was significantly reduced in spinal cords of Hpx^−/−^ mice compared with Hpx^+/+^ mice after SCI. Furthermore, the Hpx-induced M2 polarization of microglia may preserve the viability of cultured OPCs and promote their maturation. Therefore, the Hpx-induced M2 polarization of microglia may play an indispensable role in protecting myelin and promoting the regeneration process after SCI.

LRP-1 triggers different cell responses by recruiting different ligands. For instance, LRP-1 is associated with endocytosis in CNS cells^40^, has major roles in transporting cholesterol-associated lipoproteins through blood–brain barrier and subsequent metabolism^41^, modulating oligodendrogenesis^42^, and modulating the integrity of the blood–brain barrier^43,44^. Among the many ligands of LRP-1, plasminogen activator inhibitor-1 (PAI-1) was shown to modulate microglia migration^45^ and phagocytic activity^46^. We are interested in examining whether PAI-1 also plays a role in regulating microglia polarization similar to Hpx. Notably, LRP-1 is upregulated in the rims of chronic active MS lesions, where the number of M2 microglia is simultaneously increased^47^. Interestingly, an LRP-1 deficiency downregulates M2 marker expression in bone marrow and peritoneal macrophages while enhancing the macrophage response to M1 stimuli^48^, although the ligand is unclear. Thus our findings further expanded the results from previous studies and showed that the LRP-1 pathway is required for the M2 polarization of microglia by recruiting Hpx.

In summary, the data presented here reveal a novel function for Hpx, an acute-phase plasma glycoprotein, in the regulation of microglia polarization and suggested potential therapeutic benefits of Hpx in alleviating the secondary injury and improving functional repair after SCI.

## Materials and methods

### Animal experiments

All animal experiments were approved by the Second Military Medical University Committee on Animal Care. Hpx-deficient mice (Hpx^−/−^ mice) were kindly provided by Dr. Raymond F. Regan (Dept. of Emergency Medicine, Thomas Jefferson University, Philadelphia, USA)^49^ and were originally produced by Dr. Emanuela Tolosano (University of Turin, Italy)^50^. The mice were bred with wild-type B6;129 mice and maintained under normal conditions. Genotyping primers were: Hpx^−/−^ and Hpx^+/+^ (forward, TCCTGTGTGGCCTTTGCAGC; reverse, GATGCGGTGGGCTCTATGGC; CAACTTCGGCAACTCTCCCG, 220- and 190-bp bands were observed for Hpx−/− and Hpx+/+, respectively). PCR conditions were: 94 °C for 4 min; 35 cycles of 94 °C for 30 s, 56 °C for 30 s, and 72 °C for 30 s; followed by 72 °C for 10 min. The CAG-eGFP mice were purchased from the Nanjing Biomedical Research Institute of Nanjing University (Nanjing, China) and maintained under normal conditions.

### Cell culture

CNS mixed glial cell cultures were generated from the cerebral cortex of postnatal (~24 h old) mice and cultured in Dulbecco’s modified Eagle’s medium/F12 containing 10% fetal bovine serum (GIBCO, Australia) (D10) and an antibiotic mixture (1% penicillin/streptomycin) (GIBCO, Australia) at 37 °C and 5% CO_2_ for 10 days, as previously described^51^. Cultures were shaken for 6 h at 180 rpm at 37 °C to collect and purify microglia. Primary microglia were activated with LPS (Sigma-aldrich, St. Louis, MO, USA) to mimic the M1 polarization of microglia after SCI in vivo and then were incubated with Hpx (0.5–50 ng/mL) for 24 h.

Purified OPCs were isolated by collecting the floating fraction of 10-day-old mixed glial cultures after the depletion of microglia as described above, with a 16 h incubation on a rotary shaker at 37 °C and 210 rpm. Microglia and astrocytes were depleted by differential adhesion in uncoated petri dishes for 1 h at 37 °C and 5% CO_2_. The OPCs collected in D10 medium were then plated at densities ranging from 5000 to 50,000 cells/cm^2^ and maintained at 37 °C and 5% CO_2_. OPCs were grown in Neurobasal medium supplemented with 2% B27 (GIBCO) and 10 ng/mL biotin (Sigma-Aldrich) with 5 μg/mL *N*-acetyl cysteine (Sigma-Aldrich). OPCs were treated with microglia-conditioned media, which was added at a 1:1 ratio to OPC culture media.

Cortical neurons were dissected from E18 mouse embryos. After digestion, neuronal cells were suspended and seeded at a density of 5 × 104 cells per well. Neurons were cultured with a 1:1 ratio of Neurobasal medium/B27 and microglia-conditioned medium.

### Lentiviral transduction

The siRNAs for LRP1 (NM_008512) (LRP1i) were ligated into the GV493 plasmid (GeneChem). The sequence for the control siRNA (Ctrli) was as follows: 5′-TTCTCCGAACGTGTCACGT-3′. The sequences for LRP1 siRNA were as follows: 5′-TACCTACAAGATGTATGAA-3′, 5′-TGAACACATTCTTTGGTAA-3′, and 5′-GCGCCTGTGTGGTCAATAA-3′. Titers of concentrated viral particles were 6 × 10^8^ transducing units/mL. Lentiviral particles were added on day 2 to cultured microglia. The supernatant was removed 24 h after infection and replaced with D10 medium. qPCR analyses verified that lenti-LRP1i downregulated the level of LRP1 mRNA in microglia from Ctrli groups (MG LRP1i, 0.36 ± 0.13 vs. MG Ctrli, 1 ± 0.04; one-way analysis of variance (ANOVA) with Tukey’s post hoc test, *p* = 1.28E-8).

### RNA isolation and qPCR

Total RNA was extracted from the spinal cord or from primary cell cultures using Trizol (Invitrogen, CA, USA). First-strand cDNAs were synthesized using a RevertAid First Strand cDNA Synthesis Kit (Thermo Scientific Fermentas, Vilnius, Lithuania). qPCR was performed on a MyiQ Real Time PCR system (Bio-Rad, Hercules, CA, USA). Gene expression was expressed as the mRNA level, which was normalized to the mRNA level of a standard housekeeping gene (*Gapdh*) using the ΔΔCT method. At least three independent experiments were performed for each set of PCR analyses. The primers used in this study are listed in Supplementary Table 1.

### Spinal cord crush injury

Surgeries were performed using previously reported methods^52,53^, with some modifications. Briefly, mice were anesthetized with chloral hydrate and underwent a laminectomy at spinal cord segments T8–T9. For each mouse, a pair of forceps was used to laterally compress the spinal cord (0.4 mm thickness) and these compressions were maintained for 15 s, establishing the injury groups. A laminectomy group without crush injury was also included in the present study (Laminectomy). Bladders were expressed twice daily until the mice reached spontaneous micturition. For transplantation of green fluorescent protein (GFP)-tagged microglia in the spinal cord, a laminectomy was performed and microglia were injected in the same area using the above paradigm. In this model, microglia (1 × 10^6^ cells in 10 μL) were injected at a flow rate of 1 μL per min using a 10 μL Hamilton syringe (Hamilton, Reno, NV, USA) with a 29-gauge needle. To sustain high local Hpx protein levels in the lesion site, 1 μL recombinant adeno-associated virus serotype 9 vectors expressing mouse Hpx (NM_017371), generated by Obio Technology Co. Ltd. (China) (viral titers: 1 × 10^13^ particles/mL), were injected into the lesion site using the above paradigm during the surgery using a 2.5 μL Hamilton syringe (Hamilton, Reno, NV, USA) with a 33-gauge needle^54,55^.

### Behavioral assessment

The functional performance of hindlimbs was assessed using the BMS^56^ and swimming score^57^ at the indicated time points after SCI in a double-blind manner.

The BMS is a 10-point scale ranging from 0 to 9. It involves the following parameters and scores: (0) no ankle movement; (1) slight ankle movement; (2) extensive ankle movement; (3) plantar placing of the paw with or without weight support or occasional, frequent, or consistent dorsal stepping but no plantar stepping; (4) occasional plantar stepping; (5) frequent or consistent plantar stepping, no coordination or frequent, or consistent plantar stepping, some coordination, paws rotated at initial contact and lift off; (6) frequent or consistent plantar stepping, some coordination, paws parallel at initial contact, or frequent or consistent plantar stepping, mostly coordinated, paws rotated at initial contact and lift off; (7) frequent or consistent plantar stepping, mostly coordinated, paws parallel at initial contact and rotated at lift off, or frequent or consistent plantar stepping, mostly coordinated, paws parallel at initial contact and lift off, and severe trunk instability; (8) frequent or consistent plantar stepping, mostly coordinated, paws parallel at initial contact and lift off, and mild trunk instability, or frequent or consistent plantar stepping, mostly coordinated, paws parallel at initial contact and lift off, and normal trunk stability, and tail down or up and down; and (9) frequent or consistent plantar stepping, mostly coordinated, paws parallel at initial contact and lift off, and normal trunk stability and tail always up. The 11-point BMS subscore is used to evaluate finer aspects of locomotor capacity, which are not revealed by the BMS. BMS subscore includes the following interval of scores: plantar stepping (0–2); coordination (0–2); paw position (0–4); trunk stability (0–2); and tail position (0–1). Each mouse was habituated to the testing conditions prior to the operation. Briefly, mice were individually placed in an open field and allowed to move freely for 5 min; hindlimb locomotor recovery was assessed by measuring joint movements, stepping ability, coordination, and trunk stability. On the first day after injury, any mouse showing a BMS score >0.5 was excluded from further studies.

We used the swimming test to obtain information about locomotor performance in the absence of cutaneous and proprioceptive input from the limbs to the spinal cord, as previously reported^57^. Briefly, mice were allowed to swim in a 1 m-long and 6 cm-wide tank. Swimming performance was evaluated by scoring the following features: hindlimb movements (0–5 points), hindlimb/forelimb coordination (0–2 points), tail position (0–1 points), paw position (0–1 points), and sagittal and coronal balance (0–1 points). Each mouse was required to cross the tank twice and was assigned points in each testing session. Meanwhile, the number of forelimb and hindlimb strokes and the latency to reach the platform were also recorded^58^.

### Western blot analysis

Spinal cord tissue (1 mm above and below the lesion site, ~ 2 mm length) from the lesion site (T8–T9) was homogenized in RIPA buffer supplemented with a protease inhibitor cocktail (Roche, Mannheim, Germany). Tissue lysates were subjected to western blotting using an anti-Arg-1 (Abcam, Cambridge, MA) or anti-Hpx (Abcam, Cambridge, MA) antibody. Protein bands were analyzed and quantified using densitometry and an Image-Pro Plus analysis system (Media Cybernetics, Silver Spring, MD) by normalizing their levels to the levels of GAPDH bands.

### LFB staining

Staining was performed using a previously reported method^51^. Briefly, thoracic spinal cord tissues (T8-T9, 1 mm above and below the lesion site, ~2 mm length) from the lesion site were isolated and cut into cryosections (12-μm thick) for immunohistochemistry. One of every six successive sections was collected from each animal. One pool of the sections were stained with LFB and mounted in Permount (Fisher Scientific, Atlanta, GA). Images were captured with a bright-field microscope. Scoring for LFB-stained sections was performed by independent readers in a blinded manner. Six of thee 36 serial sections were examined per animal for each individual analysis.

### Immunofluorescence staining

Cells or tissue sections were fixed with 4% paraformaldehyde in phosphate-buffered saline, permeabilized, and incubated with primary antibodies (MBP, Millipore, Billerica, MA, USA; IBA-1, Wako, Osaka, Japan; GFAP, Abcam, Cambridge, MA, USA; TNF-α, R&D Systems, Minneapolis, MN, USA; 5-HT, Immunostar; NeuN, Millipore; NG2, Millipore; BrdU, Sigma-Aldrich) overnight at 4 °C, followed by an incubation with a TRITC-conjugated, fluorescein isothiocyanate (FITC)-conjugated or DyLight 405-conjugated secondary antibody (Jackson ImmunoResearch, West Grove, PA, USA) and counterstaining with Hoechst 33342 (Sigma-Aldrich). Fluorescence images were captured using a fluorescence microscope (DXM1200, Nikon, Japan) or a spectral scanning confocal microscope (TCS SP5, Leica Microsystems GmbH, Germany). For quantification, the outline of the coronal section was traced at 20× magnification and quantified using the Image-Pro Plus. Results are expressed as an average number of positive cells or percentage of areas per coronal section, as indicated.

Quantification of serotoninergic raphespinal sprouting was performed as described with slight modifications^59–61^. At least 30 sagittal sections (20 μm thick) per one mouse were included, and half of all sagittal sections were quantitatively determined for 5-HT immunostaining. The photographs were digitized with an Image-Pro Plus. After background correction, the fluorescent intensity of 5-HT^+^ fibers in each slide was automatically detected within 1 mm rostral and caudal to the lesion epicenter. Then the ratio of average 5-HT densities (caudal/rostral) for each slide from each animal was obtained and statistical analysis was performed.

### Cytotoxicity and apoptosis assays

The viability of OPCs was evaluated by terminal deoxinucleotidyl transferase-mediated dUTP-fluorescein nick end labeling (TUNEL) staining using an In Situ Cell Death Detection Kit (Roche), according to the manufacturer’s protocol. Fluorescence images were captured using a fluorescence microscope (DXM1200, Nikon) and the proportion of TUNEL^+^/NG2^+^ cells of the total cells was quantified using Image-Pro Plus.

### Isolation of microglia derived from the spinal cord

The spinal cords of SCI mice (T8–T9) were isolated, homogenized, filtered centrifuged, and then suspended in 70% Percoll (GE Healthcare, Uppsala, Sweden) and overlaid with 37 and 30% gradients. After centrifugation, the majority of mononuclear cells, which were located at the interface of 37 and 70% Percoll, were collected. These cells were incubated with a mouse FITC-labeled CD16/CD32 antibody (eBioscience, San Diego, CA, USA) for 5 min and then surface stained with allophycocyanin-labeled CD45 (eBioscience) and phycoerythrin (PE)-Cy7-labeled CD11b antibodies (BD Biosciences, San Jose, CA, USA), according to the manufacturer’s instructions. After permeabilization, cells were then stained with an Arg-1 antibody (BD Biosciences, San Jose, CA, USA), followed by a PE-labeled goat anti-mouse secondary antibody. Arg-1^+^/CD45^+^/CD11b^+^ cells were then assessed and sorted on a Moflo XDP instrument (Beckman Coulter, Brea, CA, USA).

### Statistical analysis

Data were analyzed using Student’s *t*-test to compare two groups and one-way ANOVA with Tukey’s post hoc test to compare multiple groups. The demyelinated areas from two groups were compared using the non-parametric Mann–Whitney test. The behavioral tests were analyzed using a generalized linear model with generalized estimating equations to compare four groups. The relationship between spared white matter and final BMS score of each mouse was assessed with Pearson’s regression analysis. Data are presented as means ± SEM, unless indicated otherwise. *p*-Values of <0.05 were considered statistically significant.

## Electronic supplementary material

Supplemental Material 1

Supplementary Figure Legends

S1

S2

S3

S4
