# Supplementary material for: Plasma Hemopexin ameliorates murine spinal cord injury by switching microglia from the M1 state to the M2 state
Source: Cell Death Dis. 2018 Feb 7;9(2):181. doi: 10.1038/s41419-017-0236-8 (PMC5833847; doi:10.1038/s41419-017-0236-8)
Supplement: Supplementary file 1 — Supplemental Material 1 [file 41419_2017_236_MOESM1_ESM.docx]

**Supplementary Table 1.** Oligonucleotide sequences used in this study:

| Gene | Sequence of primers |
| --- | --- |
| *Hpx* | Sense 5'TTCTTCCAAGGTAACCGCAAG 3'  Anti-sense 5'GTGCAATTCCCAACAGTTGACC 3' |
| *Tnfα* | Sense 5' GCCTCCCTCTCAGTTCT 3'  Anti-sense 5' ACTTGGTGGTTTGCTACGAC' |
| *Gapdh* | Sense 5' AAATGGTGAAGGTCGGTGTG 3'  Anti-sense 5' AGGTCAATGAAGGGGTCGTT3' |
| *Il1β* | Sense 5'TTCAGGCAGGCAGTATCA 3'  Anti-sense 5'GTCACACACCAGGTTAT3' |
| *iNos* | Sense 5'TTGACGCTCGGAACTGTAG 3'  Anti-sense 5' GACCTGATGTTGCCATTGT 3' |
| *Arginase 1* | Sense 5'GCTTGCTTCGGAACTCAAC 3'  Anti-sense 5' CGCATTCACAGTCACTTAGG 3' |
| *Lrp1* | Sense 5' CGGAGTCACTTACATCAATAATCGT 3'  Anti-sense 5' GCCATTCACATTTCTTGCGGTCA 3' |
