# Supplementary material for: Plasma Hemopexin ameliorates murine spinal cord injury by switching microglia from the M1 state to the M2 state
Source: Cell Death Dis. 2018 Feb 7;9(2):181. doi: 10.1038/s41419-017-0236-8 (PMC5833847; doi:10.1038/s41419-017-0236-8)
Supplement: Supplementary file 2 — Supplementary Figure Legends [file 41419_2017_236_MOESM2_ESM.docx]

**Supplementary Figure Legends**

**Supplementary Figure 1.** Fewer M1 microglia transplanted into the spinal cord of Hemopexin knockout mice showed conversion to the M2 phenotype. (A) Schematic diagram of the microglia transplantation experiments. Microglia from CAG-eGFP mice were pretreated with LPS for 24 h, transplanted into T8-9 of the spinal cord of Hpx^+/+^ or Hpx^-/-^ mice, and examined using immunostaining. (B) Representative micrographs showing that some GFP^+^ microglia in the spinal cord were Arg-1^+^ (red) 3 days after transplantation. (C) Percentage of Arg-1^+^ microglia of the transplanted GFP^+^ microglia. N=5 mice per group. *** p<0.01* compared with the indicated control. Scale bar=20 µm.

**Supplementary Figure 2.** Assessment of spared myelin in the spinal cord after injury. Representative micrographs of LFB staining showing spared myelin in the spinal cord of Hpx^+/+^ mice (A and B) and Hpx^-/-^ mice (C and D) on 7 dpl. (A-D) Boxed areas in the upper panel are enlarged in the lower panel. (E) Quantification of the spared myelinated areas of the spinal cord. (n = 6 mice per group). **p*<0.05, ***p*<0.01 compared with the indicated control. Scale bar=100 µm.

**Supplementary Figure 3.** Assessment of neuronal survival in the spinal cord after injury. Representative micrographs of NeuN immunostaining show the spared ventral horn neurons in Hpx^+/+^ mice (A and B) and Hpx^-/-^ mice (C and D) after SCI. The boxed areas in the left column (A-D) are shown at a higher magnification in the right column (a-d). (E) Quantification of spinal cord neuron survival at (A and C) and 0.8 mm rostral (B and D) to the epicenter. (F) Quantification of ventral horn neuron survival at (A and C) and 0.8 mm rostral (B and D) to the epicenter. **p<*0.05*, **p <*0.01*, ***p <*0.005 compared with the indicated control. Scale bar, 100 µm. N=6 per group.

**Supplementary Figure 4.** The effect of the CM from microglia on the viability of cultured neurons. (A-E) Neurons were incubated with conditioned medium (CM) from MG^LPS^, MG^LPS+HPX^, MG^Hpx^ or unstimulated microglia (control) for 48 h and then analysed using the TUNEL assay. (F) Quantification of the percentage of neuronal apoptosis using the TUNEL assay. Data are presented as the means ± SEM of three independent experiments. ****p*<0.005 compared with the MG^LPS^ group. Scale bar=40 µm.
