# Supplementary figures and images for: Plasma Hemopexin ameliorates murine spinal cord injury by switching microglia from the M1 state to the M2 state
Source: Cell Death Dis. 2018 Feb 7;9(2):181. doi: 10.1038/s41419-017-0236-8 (PMC5833847; doi:10.1038/s41419-017-0236-8)

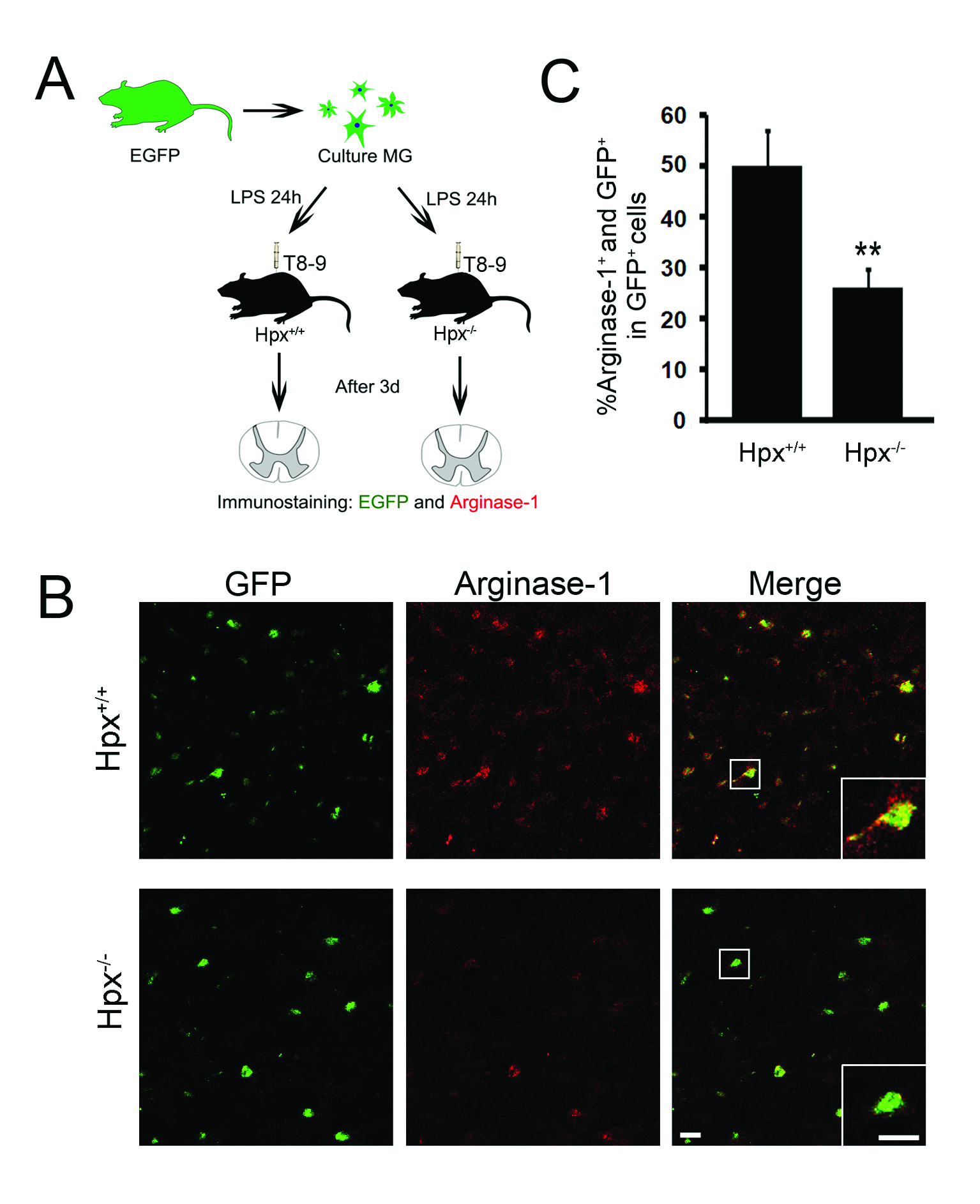

Supplement: Supplementary file 3 — S1 [file 41419_2017_236_MOESM3_ESM.tif]

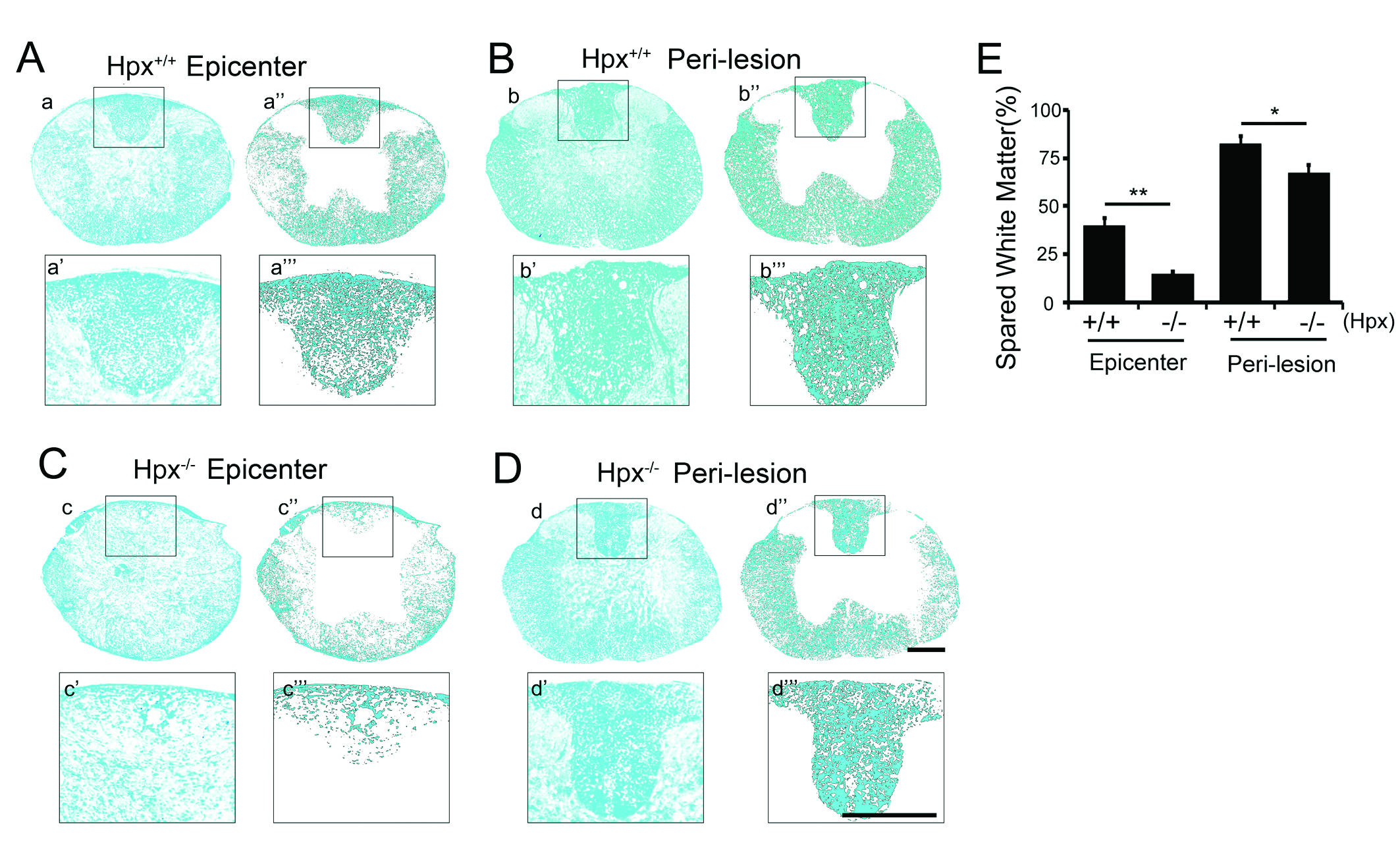

Supplement: Supplementary file 4 — S2 [file 41419_2017_236_MOESM4_ESM.tif]

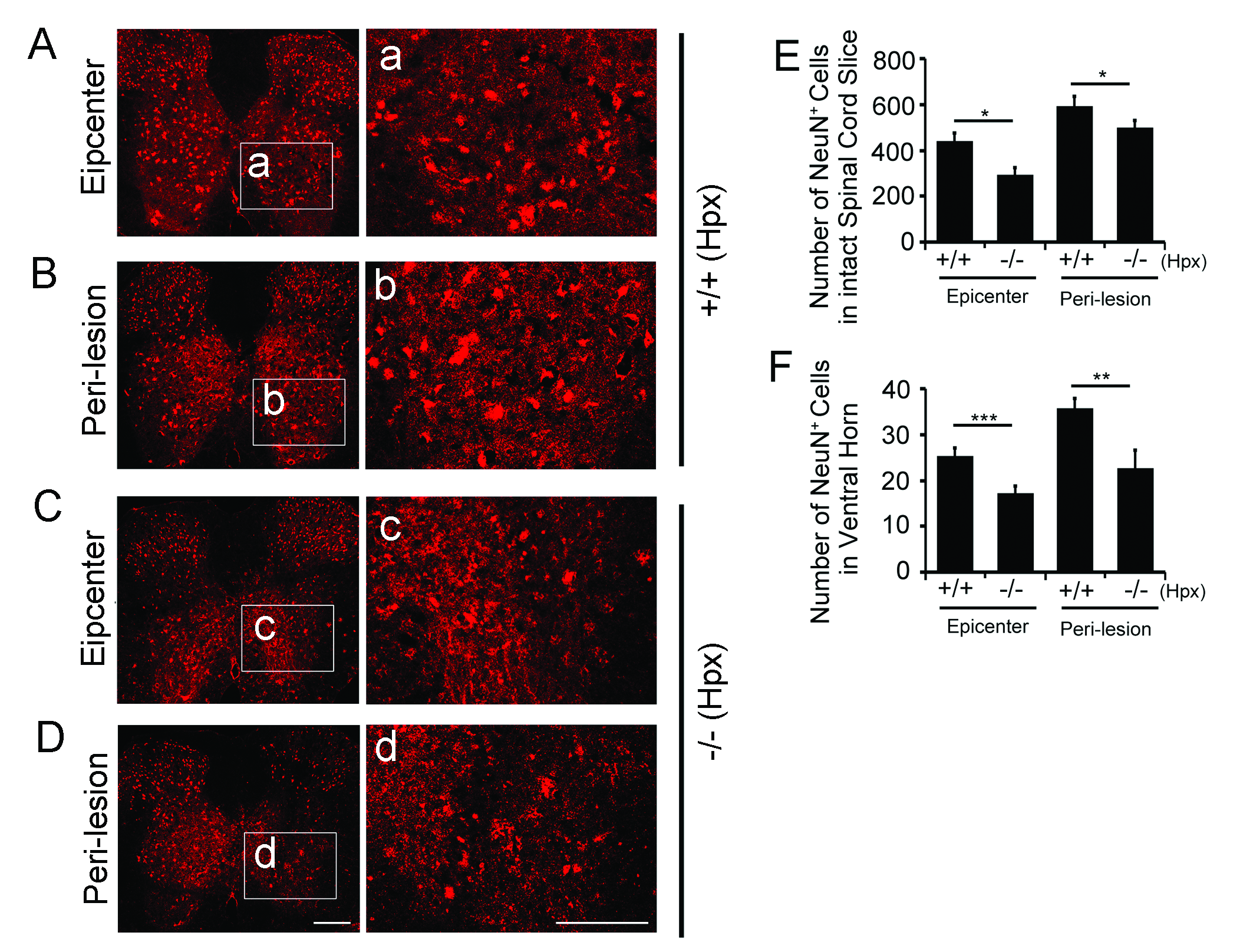

Supplement: Supplementary file 5 — S3 [file 41419_2017_236_MOESM5_ESM.tif]

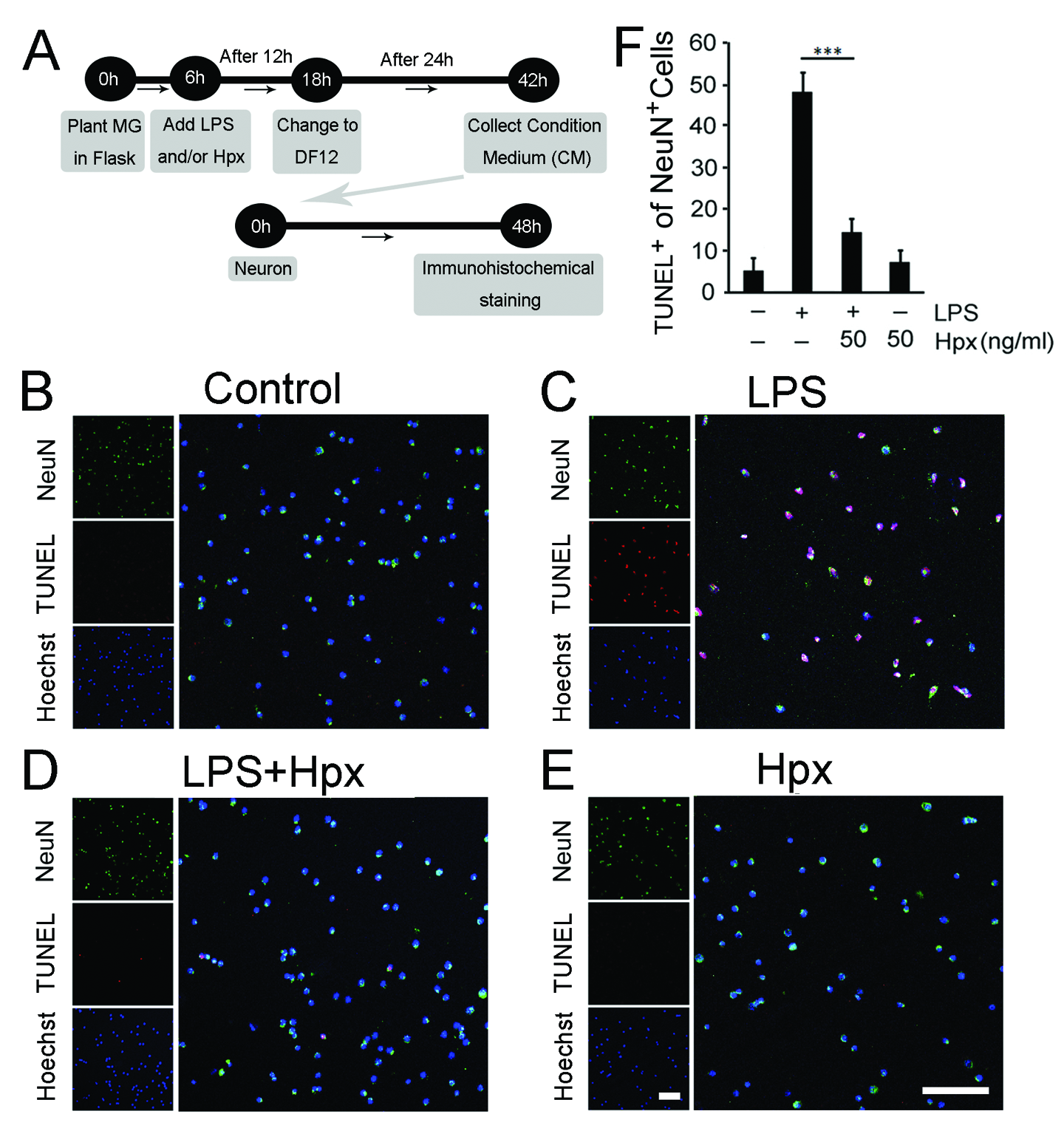

Supplement: Supplementary file 6 — S4 [file 41419_2017_236_MOESM6_ESM.tif]
